# Patient-Preferred Prosthetic Ankle-Foot Alignment for Ramps and Level-Ground Walking

**DOI:** 10.1109/TNSRE.2020.3033711

**Published:** 2021-02-25

**Authors:** Max K. Shepherd, Ann M. Simon, Joey Zisk, Levi J. Hargrove

**Affiliations:** Department of Biomedical Engineering, Northwestern University, Evanston, IL 60208 USA, also with the Neurobionics Laboratory, University of Michigan, Ann Arbor, MI 48109 USA, and also with the Center for Bionic Medicine, Shirley Ryan AbilityLab (formerly, RIC), Chicago, IL 60611 USA. He is now with (Google) X, Mountain View, CA 94043 USA.; Center for Bionic Medicine, Shirley Ryan AbilityLab (formerly, RIC), Chicago, IL 60611 USA, and also with the Department of Physical Medicine and Rehabilitation, Northwestern University, Evanston, IL 60208 USA.; Prosthetics and Orthotics, Shirley Ryan AbilityLab, Chicago, IL 60611 USA.; Center for Bionic Medicine, Shirley Ryan AbilityLab, Chicago, IL 60611 USA, also with the Department of Biomedical Engineering, McCormick School of Engineering, Technological Institute, Northwestern University, Evanston, IL 60208 USA, and also with the Department of Physical Medicine and Rehabilitation, Northwestern University, Chicago, IL 60611 USA.

**Keywords:** Prosthetics, perception, biomechanics

## Abstract

Patient preference of lower limb prosthesis behavior informally guides clinical decision making, and may become increasingly important for tuning new robotic prostheses. However, the processes for quantifying preference are still being developed, and the strengths and weaknesses of preference are not adequately understood. The present study sought to characterize the reliability (consistency) of patient preference of alignment during level-ground walking, and determine the patient-preferred ankle angle for ascent and descent of a 10° ramp, with implications for the design and control of robotic prostheses. Seven subjects with transtibial amputation walked over level ground, and ascended and descended a 10° ramp on a semi-active prosthetic ankle capable of unweighted repositioning in dorsiflexion and plantarflexion. Preferred ankle angle was measured with an adaptive forced-choice psychophysics paradigm, in which subjects walked on a randomized static ankle angle and reported whether they would prefer the ankle to be dorsiflexed or plantarflexed. Subjects had reliable preferences for alignment during level-ground walking, with deviations of 1.5° from preference resulting in an 84% response rate preferring changes toward the preference. Relative to level walking, subjects preferred 7.8° (SD: 4.8°) of dorsiflexion during ramp ascent, and 5.3° (SD: 3.8°) plantarflexion during ramp descent. As the ankle angle better matched the ramp angle, socket pressures and tibial progression (shank pitch) both more closely mirrored those during level walking. These findings provide baseline behaviors for prosthetic ankles capable of adapting to slopes based on patient preference, and provide strong evidence that people with transtibial amputation can finely perceive ankle alignment.

## I. Introduction

QUALITY of life for individuals with unilateral transtibial (TT) amputations may be impacted by proper alignment of prosthetic componentry. It is difficult to definitively link long-term secondary health consequences to specific misalignments, but poor alignment may lead to increased or asymmetric joint forces [1]–[3], which can in turn have deleterious effects on joint structures long-term, particularly in the back and unaffected limb [4]. Poor alignment may also induce excessive shear and normal forces in the residuum-socket interface, leading to issues with skin breakdown [5]. Unique residuum and socket geometries add to the difficulty, and make alignment difficult to truly codify. Many guidelines exist to arrive on proper alignment via visual assessment, and prosthetists are trained to identify common gait deviations and make adjustments expected to mitigate them [6]–[8].

Incline walking presents a particular challenge for individuals with TT amputations, in terms of comfort and safety. Internal stresses in the muscle flap covering the truncated tibia have been found to increase by 50% during ramp descent [9], socket moments are increased [10], [11], and kinematics are highly asymmetric [12]. A relatively new class of semi-active ankle-foot prostheses are capable of microprocessor-controlled modification of mechanics based on task. For instance, Ossur’s PROPRIO FOOT incorporates an ankle joint capable of realignment of the foot when unloaded [13]. These semi-active feet are particularly promising in adapting to tasks beyond level-ground walking. Specifically, aligning the foot to more closely match the ramp angle (*i.e*., dorsiflexing the foot during ramp ascent and plantarflexing during ramp descent) reduces socket pressures and compensatory mechanisms [11], [14], [15]. Similar results have been found during stair ascent and descent [11]. It is also possible that modifying the ankle angle could improve the energetic cost of ramp ambulation, as modifying shoe outsole geometry to better match ramp angle has been found to reduce metabolic cost of walking on ramps in healthy adults [16].

With this new class of semi-active feet, the number of adjustable variables expands, and there are no rigorous methods for selecting the appropriate behaviors (e.g., the appropriate ankle angle for ramp ascent). Furthermore, many of the behaviors are pre-programmed and not adjusted on an individual-specific basis, placing more responsibility on manufacturers to select appropriate default behaviors and determine variables which should be tuned by the prosthetist during the fitting and dynamic alignment process.

Patient preference may be one of the most immediately relevant outcome metrics and determinants of optimal device behavior. Prosthetists often rely on communication with patients to select and align componentry, especially given that kinetic changes are difficult to visually assess, and kinematic changes are more unpredictable [17]. Patient preference has been studied, but typically involves classifying anecdotal responses or low-resolution subjective rating scales [18]. Robotic tools, including semi-active prostheses, have made investigations of patient preference more feasible, as they can quickly simulate a range of passive mechanics [19], [20]. New studies have also looked at ways to optimize wearable device behaviors over multiple parameters simultaneously based on user preference [21].

Our study has two primary motivations. First, we want to understand the ability of individuals with TT amputations to develop and communicate their preference regarding prosthetic foot alignment during normal level-ground walking. Specifically, we want to understand the short-term reliability of their preferred alignment to provide clinicians with a better understanding of the strengths and limitations of incorporating their feedback. Second, we want to determine what subjects consider to be the optimal adjustments to alignment for ramps. This information will have specific applications in the design and control of both passive and semi-active prosthetic ankles capable of automatically adjusting alignment to inclines. To answer these questions, we used a semi-active ankle to vary foot alignment while subjects walked on level-ground and ramps, providing feedback as to the adjustments they would prefer us to make.

## II. Methods

### A. Adaptive Ankle Prosthesis

**Fig. 1.**
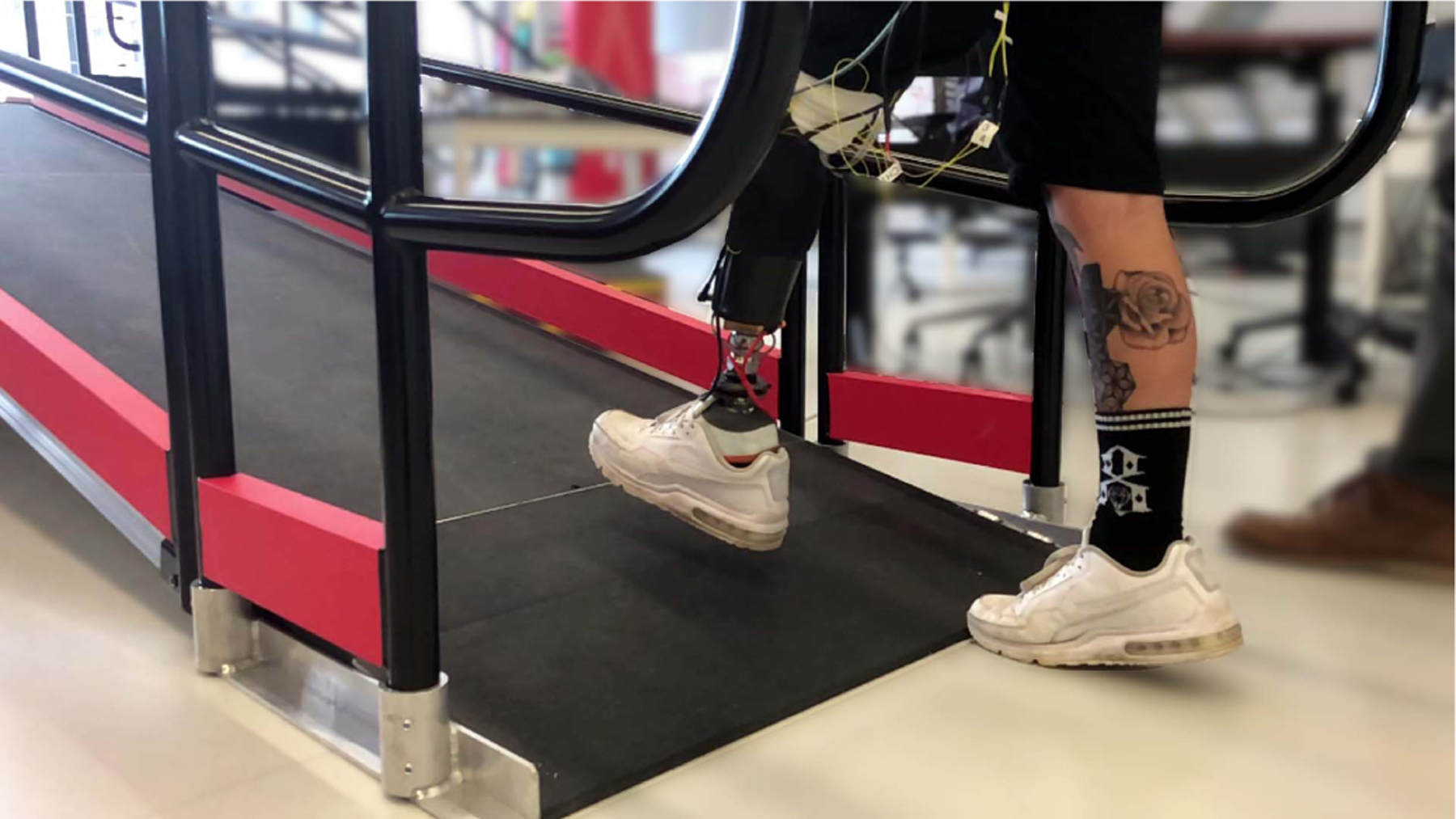
Subject with TT amputation walking with the Adaptive Ankle, dorsiflexed from its neutral angle for ramp ascent. For the experiment, subjects wore their prescribed socket and customary footwear.

The ankle component used in this study is capable of actuated dorsi/plantar flexion when unweighted, and has been previously described in detail [22] (Fig 1). Briefly, a small motor in the ankle is coupled to an ankle axis through a non-backdrivable transmission, allowing active modification of the ankle angle in the sagittal plane. It is semi-active; *i.e*., it is not designed to produce net positive work. The location of the ankle axis is similar to the location of the pyramid adaptor on many commercial feet, and thus modifying ankle angle provides a reasonable approximation to changing foot alignment for most standard prosthetic feet. The ankle was modified from the previously published version to reduce weight and size (build height), increase mechanical safety factors, improve position control speed and accuracy, and include fully enclosed embedded electronics, including Wi-Fi. The device weighs1.2 kg with a footshell and a six-axis load cell. An Ossur Variflex LP (low-profile) carbon-fiber footplate is attached below the ankle joint, with categories 5 or 8 used in this study, based on subject height. At the time of the study, the ankle mechanism had approximately 1° of backlash, and 40° range of motion. From this point, we will refer to the combination of the repositionable ankle joint and carbon-fiber footplate as the Adaptive Ankle.

### B. Subjects

Eight individuals with TT amputation (7 male, 1 female; average weight: 81.5 kg) were recruited for the study. All subjects provided written informed consent, and this study was approved by the Northwestern University Institutional Review Board (ID: STU00209522; 3/26/2019). All subjects were Medicare Functional Classification Level K3/K4 ambulators, and used a dynamic energy storage and return prosthetic foot with their prescribed prosthesis. Subject information is recorded in Table I, with residual limb length qualitatively assessed by a prosthetist on the research team. One subject did not complete the full experiment, and several subjects required small modifications to the methods; these details are in the Appendix.

### C. Fitting, Familiarization, and Subjective Evaluations

Prior to fitting, a researcher inserted force-sensing resistors (FSRs) into anterior distal, anterior proximal, posterior distal, and posterior proximal locations in the subject’s prescribed home-use socket. These sensors (FlexiForce A201 by Tekscan; Boston, USA) measured pressure at the socket / residual limb interface. The locations were chosen based on our assumption that they would sustain large pressures due to sagittal plane socket moments and for consistency with the literature [11], [23]. The anterior proximal and anterior distal FSR placements were determined by the location of the patellar tendon and anterior distal tibia, respectively, while posterior FSR placements were height-matched to the anterior sensors. A prosthetist then fit and dynamically aligned all subjects with the Adaptive Ankle (set to a constant angle), which was attached to the subject’s socket. After the alignment was determined, it was not adjusted during the rest of the experiment, and efforts were made to hold it constant between experimental sessions. Subjects walked on the ankle for a short period to familiarize themselves with the device. Ankle angle was slowly changed, and subjects were informed of these changes using the terms “toes up” or “toes down” (e.g., the experimenter might comment “we are going to bring the toes up now.”)

After subjects felt comfortable during level ground walking with the ankle positioned at different angles, subjective evaluations of socket comfort [24] and exertion were administered at seven equally spaced angles (−4.5°, − 3.0°, − 1.5°, 0°,1.5°, 3.0°, 4.5°, with negative values indicating plantarflexion). Specifically, for the socket comfort evaluation, subjects were asked: “on a 0 – 10 scale, if 0 represents the most uncomfortable socket fit you can imagine, and 10 represents the most comfortable socket fit, how would you score the comfort of the socket fit of your artificial limb at the moment?” The custom exertion score was designed to mirror the socket comfort score. The evaluations were completed twice at each angle, and the order of the tested angles was randomized. Because our subjects were skewed to higher levels of mobility than the overall population of people with TT amputation, we encouraged several to lower the average score they gave their socket comfort and exertion to make the scale more sensitive; thus while the trends are likely to be accurate, the absolute numbers on the scale may be biased and represent higher comfort or exertion ratings.

Next, this process of familiarization and subjective evaluation was completed for Ramp Ascent (RA) and Ramp Descent (RD). The ramp used in this study was 4 m long and set to a 10° incline. We tested seven angles of dorsiflexion for RA (0°, 1.5°, 3.0°, 4.5°, 6.0°, 7.5°, 9.0°) and seven angles of plantarflexion for RD (0°, −1.5°, −3.0°, −4.5°, 6.0°, − 7.5°, −9.0°).

During all trials, axial force through the pylon was measured with a 6 degree of freedom load cell (Sunrise Instruments, M3554E). A six-axis inertial measurements unit (MPU-9250) was placed anteriorly on the pylon, and recorded accelerations and angular velocities.

### D. Preference Testing

To obtain preferred ankle angle, we implemented a two-alternative forced-choice paradigm [25], in which subjects were presented with one stimulus (ankle angle) and asked to compare it with their internal notion of preference, developed during the first part of the study. We assume subjects follow a simple decision rule, in which they compare the tested stimulus to their preferred ankle angle, and report whether it is more dorsiflexed or plantarflexed than their preference (e.g., if it is too dorsiflexed, they respond that they would prefer “toes down.”) This method is in contrast to A-B testing or traditional two-alternative forced-choice testing, both of which require a reference stimulus (which does not exist in our scenario) and may require substantially more tested stimuli. We allowed different types of responses if subjects found a different vocabulary to be more intuitive (e.g., “prefer plantarflexion”). Subjects were not told the magnitude or direction of ankle angle change between trials.

For each of the three conditions (level-ground, ramp ascent, ramp descent), the test consisted of 35 trials. The tested angles in each condition were the same as previously mentioned for the subjective evaluations (seven equally spaced angles, centered around −4.5° for ramp descent, 0° for level walking, and 4.5° for ramp ascent.) Level-ground trials were performed first, followed by the ramp ascent and ramp descent trials, which were interleaved to avoid subject fatigue. For the first 14 trials, each of the seven unique angles was presented twice, in random order.

**Fig. 2.**
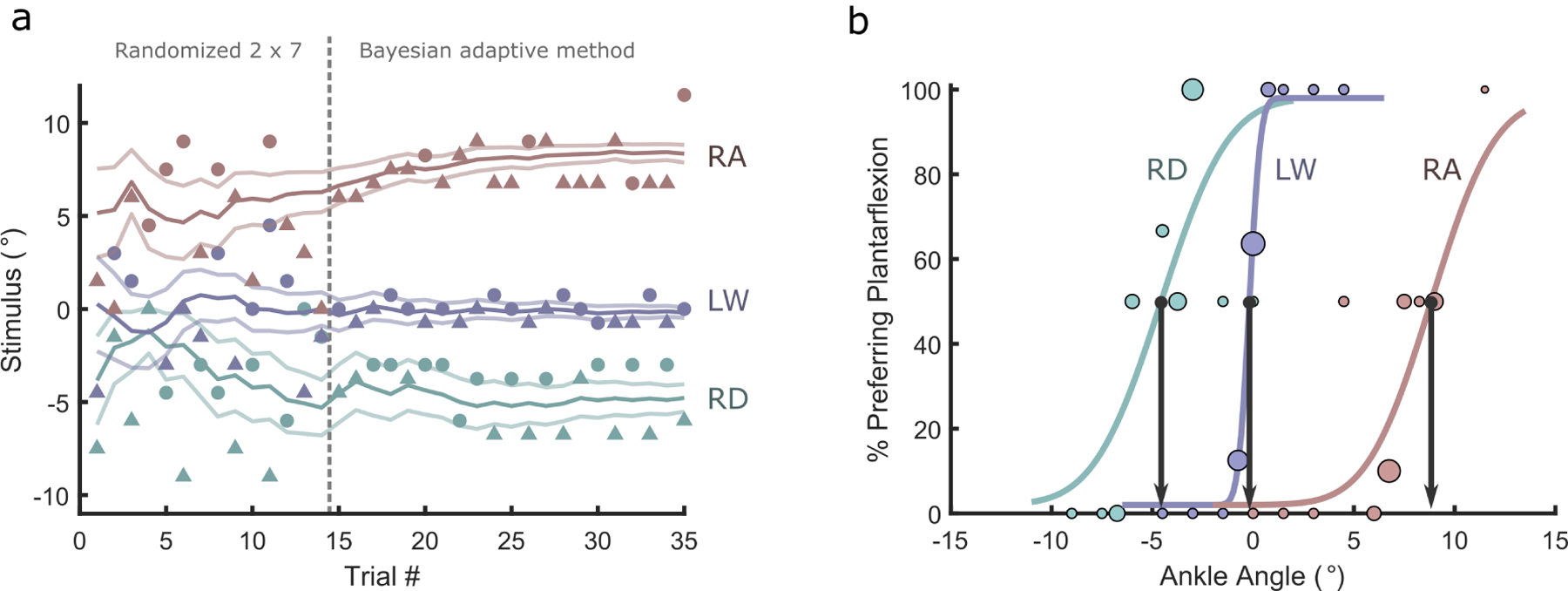
Example of preference experiment for a representative subject. a) Subjects completed 35 trials for Ramp Descent (RD), Level Walking (LW), and Ramp Ascent (RA) each. Responses indicating preference for dorsiflexing the foot from the current angle are shown with triangles, and for the foot to be plantarflexed are shown with circles. Estimated thresholds (preference) and standard errors are shown with traces. The stimuli for the first 14 trials were chosen randomly from a 2 × 7 block, with the remaining stimuli chosen by a Bayesian adaptive procedure intending to maximally improve estimates of threshold and slope (reliability). b) Fitted cumulative normal functions to the preference data (dorsiflexion is positive). Marker area is proportional to the number of trials at that ankle angle. The ankle angle corresponding to a 50% split between preferring plantarflexion and dorsiflexion represents the preference (illustrated with arrows). The slope (steepness) of the Cumulative Gaussians is inversely proportional to the reliability. Note that for this subject, preference during RA approached the edge of the prescribed test range, and the researcher manually added trials at 11° to better estimate preference.

The first 14 trials were followed by 21 trials, where stimuli were determined based on preceding responses using a Bayesian adaptive psychophysical procedure to more efficiently estimate both the point of subjective equality (preference) and slope (reliability) [25], [26]. This method prescribes more trials near the preference, reducing the number of trials at angles far from the preference, which provide much less information. In the adaptive algorithm, the prior for point of subjective equality was set to a uniform distribution over the tested range of ankle angles, and for slope was set to a uniform prior over the log of slopes between 0.3 and 3 [25]. We elected to block randomize the first 14 trials (as opposed to performing the Bayesian adaptive method for the full experiment) to increase the amount of biomechanical data available for analysis, and to provide additional reinforcement of the concept by providing several obvious stimuli, potentially boosting subject confidence [26]. An example of a representative subject’s raw preference data through the 35 trials for the three tasks is provided in Fig. 2a.

Prior to the experiment, we performed several Monte Carlo simulations using the adaptive algorithm and a simulated subject to fine-tune the algorithm. We simulated subject responses with cumulative normal psychometric functions, using various points of subjective equality, slopes, and lapse rates (the rate at which subjects respond “incorrectly, regardless of stimulus intensity” [27]). We confirmed that using each of the seven angles in the tested range twice prior to the adaptive algorithm taking over (*i.e*., the first 14 of the 35 trials) had approximately equal accuracy in estimating threshold and slope as employing the adaptive algorithm for all 35 trials. The simulations also guided us to set the assumed lapse rate to a conservative 5% in the adaptive algorithm. The lapse rate describes the fraction of trials that the subject guesses or makes a random decision regardless of the stimulus, as a result of, for instance, a lapse in attention. Though we considered it unlikely that the true lapse rate would be this high, the running slope estimates (and thus the chosen stimuli) proved particularly sensitive to underestimation of the lapse rate. It should be noted that the assumed lapse rate and prior uniform distributions for slope and threshold were used by the adaptive algorithm in selecting stimuli during the experiment, but are not used in the final fitting of the psychometric function.

We allowed minor alterations to the protocol for several subjects based on either time, fatigue, or difficulties learning or communicating the tested variable, and these are described more thoroughly in the Appendix. Notably, Subject 3 had trouble remembering the directionality of the adjustments, and was unable to perform the standard protocol on ramps. Another subject (Subject 5) was unable to provide preference feedback and was excluded from the analysis. The protocol was also adjusted after Subjects 1 and 2 to allow half-increments of 0.75° during the adaptive trials (trials 15–35), to improve resolution for subjects with high reliability. Finally, the range of dorsiflexion during ramp ascent was shifted part-way through the experiment by 6° for Subject 1, and 2.5° for Subject 8, to accommodate their higher preferred dorsiflexion.

### E. Data Processing

Raw preference data were fit with a cumulative normal function using maximum likelihood estimation, with lapse rate set at 0.02 [25] (Fig. 2b). The preference value was defined as the point of subjective equality, *i.e*., the ankle angle at which subjects were equally likely to prefer dorsiflexion as plantarflexion. The slope of the fitted cumulative normal is the inverse of the standard deviation of the underlying Gaussian describing the difference between stimulus and internal notion of preference, both assumed to be approximately Gaussian distributions. Thus, we report this standard deviation as a “Reliability Index,” which describes consistency of preference. The Reliability Index denotes the magnitude of adjustment *away* from preference that would result in an 84% response rate preferring a change *towards* the preference. An example of deriving the preference from fitted cumulative normal functions is shown in Fig 2b. Because several subjects’ RA and RD experiments were shortened, not all slope estimates (which are the inverse of the Reliability Index) were reliable. Instead, to obtain an average Reliability Index, we centered individual subjects’ responses for RA and RD by subtracting their preferred angles for the respective tasks, and then pooled these transformed responses across the six subjects that followed the standard protocol (see Appendix). We report the average Reliability Index as the slope of a cumulative normal function fit to the pooled data.

A simple benchtop experiment with hung weights was performed to calibrate FSR resistance to pressure (kPa). Pressure data were segmented by strides using the load cell for heel strike estimation, low-pass filtered with a second-order 50 Hz forward-backward Butterworth filter, and normalized by subject weight (kg). Pressure-time integrals were calculated with time expressed as a fraction of the gait cycle using the trapezoidal rule.

To obtain estimates of shank pitch, the sagittal plane gravity vector estimate from the accelerometers was fused with integrated angular velocity via a first-order complementary filter with a time constant of 1 s.

### F. Statistical Analysis

Preferred ankle angle during RA and RD were compared with preferred ankle angle during LW using one-sided paired t-tests. Exertion and socket comfort scores for each activity were fit with post-hoc linear mixed effects models, with subject (random), ankle angle (fixed), and squared ankle angle (fixed) as predictors.

## III. Results

**Fig. 3.**
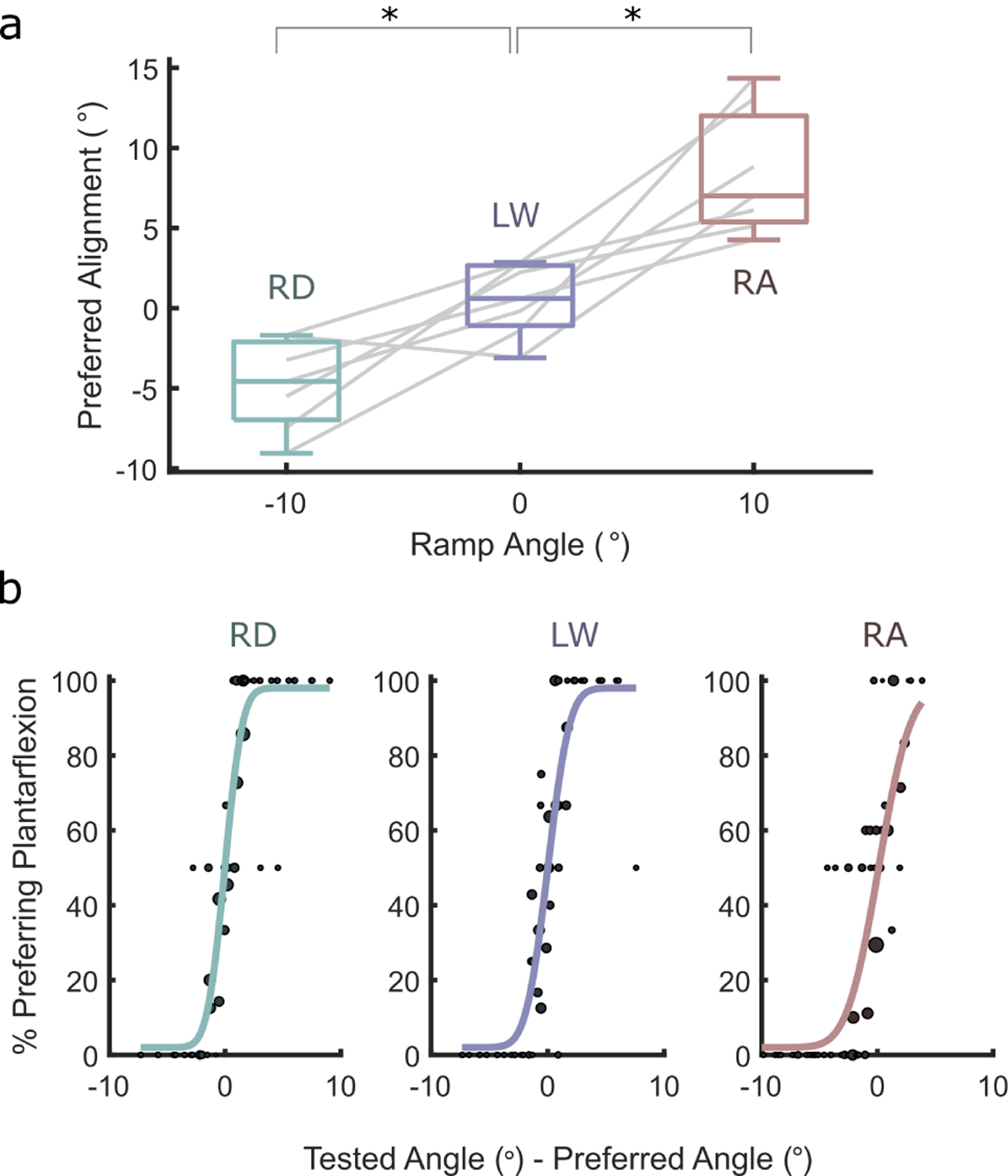
a) Preferred ankle angle vs ramp angle (*n* = 7). Positive alignment represents dorsiflexion, positive ramp angle represents ascent. Preferences for individual subjects are traced in gray. Asterisks denote paired comparisons in which *p* < 0.01. b) Comparison of consistency (*n* = 6) during ramp descent (left), level walking (middle), and ramp ascent (right) using pooled preference data. Individual subjects’ preferred ankle angles are subtracted from the stimulus levels to isolate intra-subject variability.

For all but one subject, preferred ankle angle changed as expected, with a positive correlation between ankle angle and ramp angle (Fig 3a). The mean preferred ankle angle for level-ground walking was 0.5° (dorsiflexed), with extremes of 2.9° and −3.0°, relative to the nominal alignment (the alignment set during dynamic alignment at the beginning of the experiment.)

The mean preferred ankle angle for ramp ascent was 8.4° (SD: ±3.8°) dorsiflexion, and for ramp descent was 4.7° (SD: ±2.9°) plantarflexion (Figure 3a), relative to the nominal alignment. Zeroing these angles by the subjects’ preferred angles for level walking, subjects preferred 7.8° (SD: ±4.8°) dorsiflexion for ramp ascent (*p <* 0.01) and 5.3° (SD:3.8°) plantarflexion for ramp descent (*p <* 0.01). Note the ± relatively large inter-subject variability in preferred angle for ramp ascent and descent; in particular, the range of normalized ramp ascent preferences was 2.9° to 15.7° of dorsiflexion.

The Reliability Index during LW was 1.5° (*i.e*., deviations of 1.5° from preference resulted in an 84% response rate preferring changes toward the preference.) Similarly, and in contrast to the large inter-subject variability, the Reliability Index was 1.2° for RD and 2.1° for RA (Fig 3b).

**Fig. 4.**
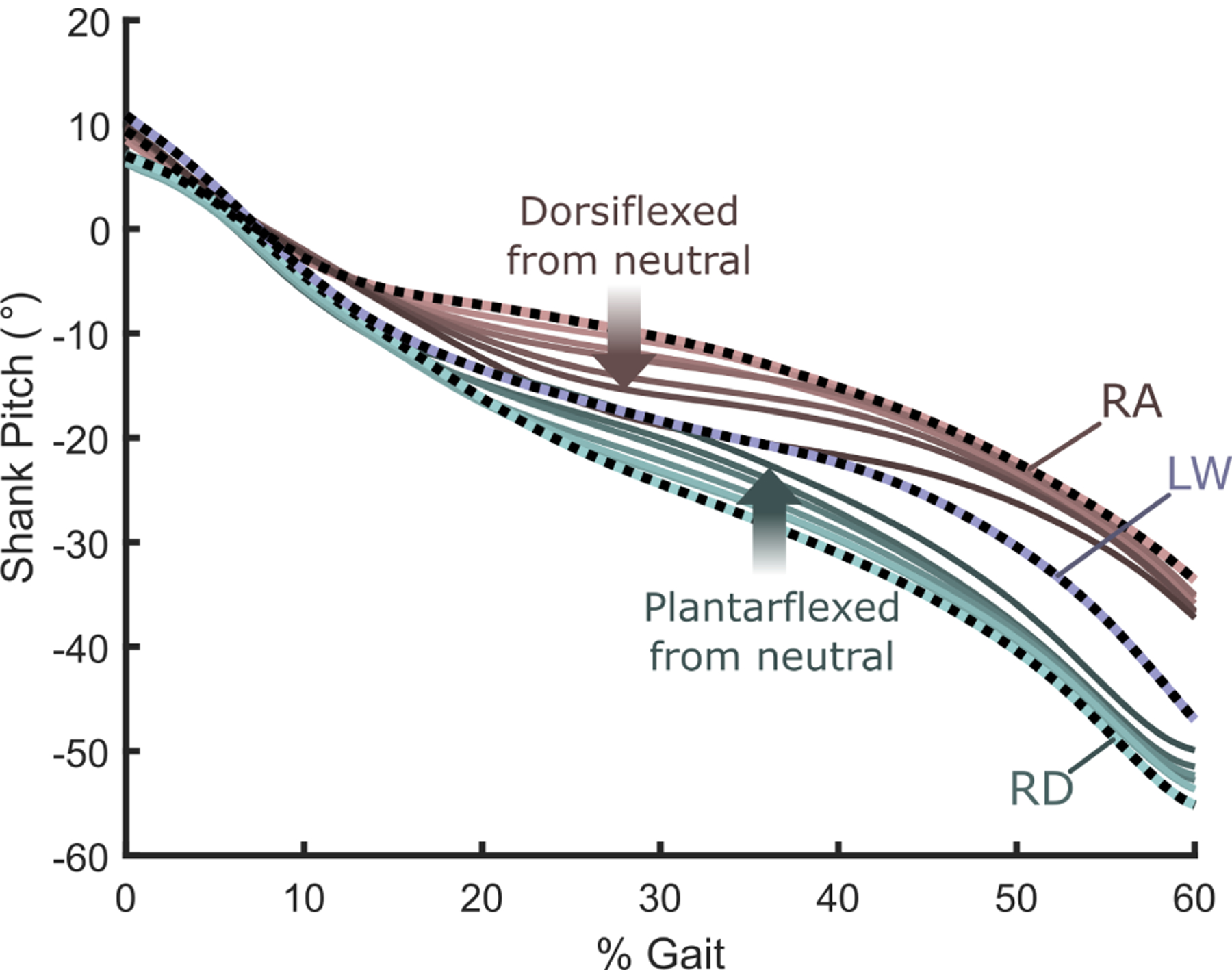
Shank pitch vs. % gait for various alignments during ramp ascent (RA) and ramp descent (RD), and for neutral alignment during level walking (LW). In RA and RD, shank pitch during midstance more closely matched shank pitch during LW as ankle angle better matched the ramp angle. For clarity, shank pitches during LW at non-neutral angles are not shown, but follow expected trends.

For both ramp ascent and ramp descent, the progression of shank pitches during stance (*i.e*., tibial progression) more closely matched that during level walking as the ankle angles approached the ramp angles (Fig. 4). Shank pitch was most sensitive to ankle angle during midstance; at approximately 30% of the gait cycle, shank pitch varied by 5.5°, 5.9°, and 8.5° during RD, LW, and RA across the tested range (9°) of ankle angles.

**Fig. 5.**
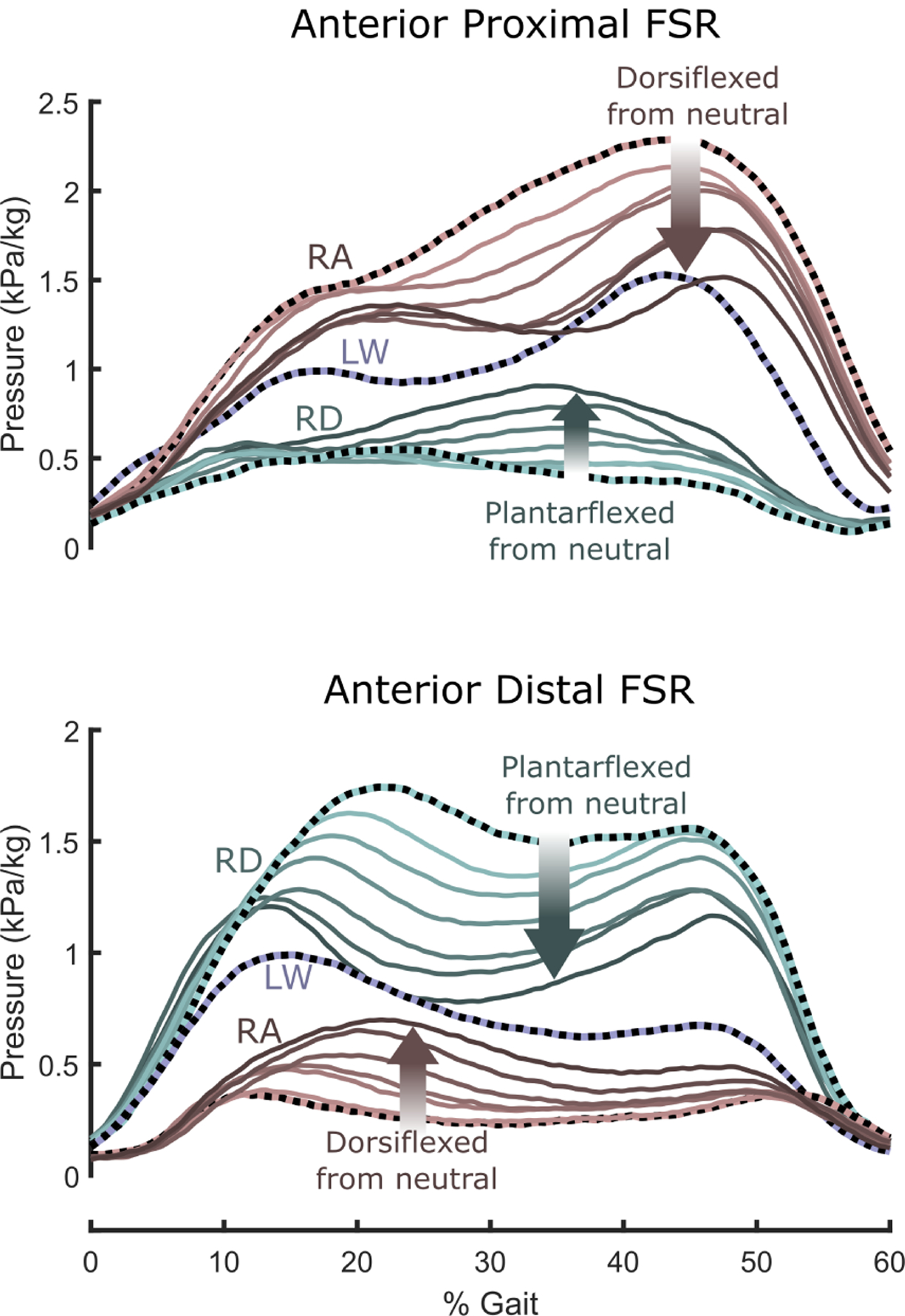
Averaged normalized socket pressures vs % gait for various ankle angles during RA (red), LW (purple), and RD (teal). Nominal alignment is shown with a dashed line, and increasing darkness denotes modification of the alignment towards the ramp angle (*i.e.*, plantarflexion during RD and dorsiflexion during RA). Socket pressure for LW is only shown at the neutral angle for clarity, but the trends are consistent and as expected. Note that as ankle angle better matches the ramp angle, the socket pressures more closely match those during level walking, and in particular, the largest pressures are reduced.

**Fig. 6.**
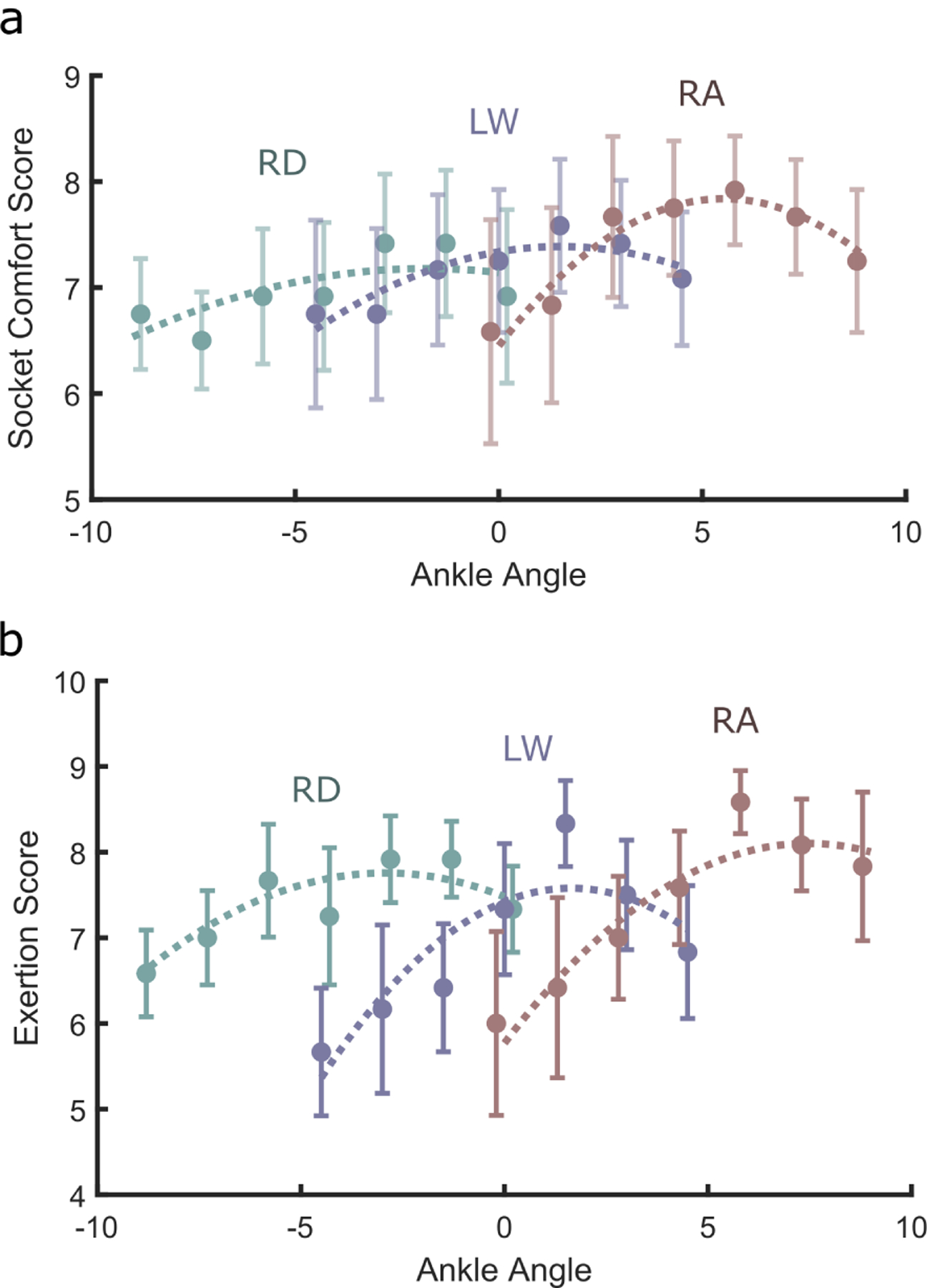
Qualitative assessments of socket comfort and exertion for various alignments during RA (red), LW (purple), and RD(teal). Error bars denote s.e.m. a) Ankle angle affected Socket Comfort Score [linear mixed effects model, RA: *p*(angle) *<* 0.01, *p*(angle^2^) *<* 0.01; LW: *p*(angle) = 0.03, *p*(angle^2^) = 0.06; RD: *p*(angle) = 0.68, *p*(angle^2^) = 0.27.] b) Ankle angle affected Exertion [linear mixed effects model, RA: *p*(angle) *<* 0.01, *p*(angle^2^)= 0.07; LW: *p*(angle)*<*0.01, *p*(angle^2^) *<*0.01; RD:*p*(angle)= 0.17, *p*(angle^2^) = 0.03].

During ramp ascent, there were large anterior proximal (patellar) pressures, which decreased as the foot was dorsiflexed (Fig. 5, top). Similarly, during ramp descent there were large anterior distal socket pressures, which decreased as the ankle was plantarflexed (Fig. 5, bottom). For both ramp ascent and ramp descent, modifying the ankle angle to more closely match the ramp angle caused socket pressure profiles to approach those during level walking, in both proximal and distal anterior locations. Despite the high sensitivity of socket pressure to ankle angle for the three tasks, however, subjects reported only mild changes in comfort on the subjective socket comfort test (Fig. 6a). These changes were most pronounced during ramp ascent, where the second order model was a strong fit [*p*(angle) *<* 0.01, *p*(angle^2^*) <* 0.01]. According to the fitted second order models, socket comfort scores peaked at −1.8°, 1.5°, and 5.5° for ramp descent, level walking, and ramp ascent respectively. Exertion followed a similar trend, with the largest effect seen in RA (Fig 6b.)

## IV. Discussion

Subjects were consistent in identifying their preferred ankle angle on level-ground (Reliability Index of 1.4°). For reference, according to a quick benchtop test, 1.4° is equivalent to approximately half a turn of a set screw with standard prosthetic componentry. A previous study employed different methods to determine a “window” of acceptability regarding prosthesis alignment, and generally found that both patients and prosthetists seem to find a large range acceptable [28]. However, there is an important difference between the notions of patient consistency and range of acceptable device behavior. Understanding the range of acceptable device behaviors can give perspective on the importance to the patient of fine-tuning, whereas patient consistency may indicate the usefulness of their feedback.

In RA and RD, both socket pressure and shank progression profiles more closely matched those of level walking as the ankle angle was aligned closer to the ramp angle (i.e., as the ankle was dorsiflexed during RA and plantarflexed during RD). Peak socket pressures were greatly reduced; dorsiflexion lowered the peak anterior proximal socket pressures during ramp ascent by ~40%, plantarflexion lowered peak anterior distal socket pressures by ~50% during RD. However, though there were significant second-order trends, the relatively high socket comfort scores across ankle angles indicated that socket pressures may not have been a large contributor to preference.

The moderately high between-subjects variability of preferred ankle angle for ramp walking suggests that ideally, the scale factor between ramp angle and ankle angle would be tuned on an individual basis. However, universally applied ankle angles for RA and RD may still provide substantial benefits, even if they do not perfectly match a patient’s true preference. Though we only tested two ramp angles, it’s possible to assume an approximately linear relationship between ramp angle and preferred ankle angle, given the relatively linear effects ramp angle has on kinematics and kinetics [29]. The results of this study provide baseline information for how semi-active devices should behave on ramps, and indicate that patients perceive they would benefit from this functionality. These results could also inform the design and recommended alignment of fully passive feet capable of automatically adapting to sloped surfaces [30], [31].

Several of the subjects struggled to develop intuition regarding the tested variable, and particularly had trouble mapping the vocabulary to sensations. Part of this confusion may be due to angle being an interval variable, in which differences between values have meaning but a true notion of “zero” doesn’t exist. Testing on inclines provided a further hindrance, as the interleaved experimental design required subjects to switch between angles that were generally plantarflexed from neutral and angles that were generally dorsiflexed from neutral. Despite subjects having high consistency once trained, our experience with the training suggests that this form of preference-gathering may not be clinically viable with this variable. We believe that other ways to find preference may have more clinical potential, such as two-stimulus comparisons (e.g., patients are asked to choose between “A” and “B”), or methods in which clinicians “hand patients the dial,” giving them control over device behavior without the need for learning a new vocabulary. For this specific study, we sought a higher accuracy of preference than would clinically be needed, and chose our specific methods to reduce the number of trials to prevent fatigue or skin breakdown issues.

Based on the larger change in preferred angle during RA (7.8°) than RD (−5.3°), and the improved socket comfort score (Fig. 5b), the angle adjustments may have provided larger benefits during ramp ascent than ramp descent. This may be because the normal role of the ankle during ramp descent is to perform negative work, enabling smooth lowering of the center of mass during stance. Though the shank angle looks more normative with increased plantarflexion (Fig. 4), the plantarflexed toe may have caused the center of mass to rise near toe off, forcing subjects to absorb substantial energy during ground contact with their sound limb. Semi-active devices capable of dissipating energy may provide better performance on ramp descent.

While these results provide initial understanding for what ankle alignments people with TT amputation prefer during level walking and ramps, there were several limitations to this study. The subject pool was small and biased towards highly active users. As previously mentioned, it was also difficult to train subjects to understand and communicate their preference regarding alignment, without a reference value for comparison (as would be present in A-B testing). As such, major alterations to the protocol had to be made for one subject during ramp testing, and minor alterations to the range of tested angles and number of trials were made for several other subjects. Subjects may have continued to adapt and their preferences changed throughout the experiment, particularly when experiments spanned multiple days. How preference changes over longer time scales is an important area of future work. Finally, the high mobility required for subjects to complete the experiment meant it was not a representative sample of the unilateral transtibial amputee population. It is possible that the overall population preferences are biased away from those found in this experiment, and consistencies are not as high.

Intent recognition methods are capable of predicting transitions onto ramps using onboard sensors prior to the first step [32]. After the first heel-strike, the ramp angle could then be accurately measured using onboard inertial measurement units and load cells, via estimations of the gravity vector and deflection of the foot. Thus, it is possible that a small adjustment can be made to the angle prior to the first step based on intent recognition, and the ankle can refine this once prior to the second step.

## V. Conclusion

In this work, we used a semi-active prosthetic ankle capable of angular adjustments to determine what changes to alignment prosthesis users prefer, and measure biomechanical outcomes that may give insight into the factors behind their preference. Despite difficulties in training subjects to communicate desired directional changes to alignment, subjects exhibited highly repeatable preferences (1.5° during level walking). As expected, subjects preferred the foot to be dorsiflexed during ramp ascent and plantarflexed during ramp descent. Potential factors of preference include tibial progression that looks similar to level walking as ankle angle better matched the ramp angle, and decreasing in peak anterior socket pressures.

## Figures and Tables

**TABLE I:** Subject Information

Subject Number	Weight (kg)	Sex	Suspension	Residual Limb Length	Prescribed Prosthesis
1	53	F	Pin-lock	short	Elation, by Ossur
2	88	M	Pin-lock	short	Proflex Pivot, by Ossur
3	88	M	Pin-lock	long	Velocity, by College Park
4	61	M	Pin-lock	short	Proflex LP, by Ossur
5	86	M	Suction	average	Sierra, by Freedom Inn.
6	98	M	Vacuum	short	Proflex XC, by Ossur
7	88	M	Vacuum	average	Meridium, by Ottobock
8	90	M	Suction	average	Vari-flex, by Ossur

## Appendix

Subject 3 became confused about the language after the adaptive algorithm homed in on his preference for an extended number of trials. This was confirmed when the ramp trials began. We decided to remove the final eight trials, which appear clearly demarcated in terms of response. There is a degree of arbitrariness to this cutoff; accordingly, we have decided to exclude his data from analysis of the Reliability Index, which would be much more heavily biased than the preference by the exclusion of the last eight trials. To determine his preference on ramps, we implemented a method of adjustment paradigm, in which the subject verbally requested a change to the ankle angle, and we implemented the requested change. The advantage of this method is that the subject could feel the change immediately following the request, constantly reinforcing the mapping between requested changes and perceived changes, and quickly catching and correcting errors if they occurred.
